# P-1433. Characterization and Georeferencing of Tuberculosis Cases in Pediatric Patients at a Reference Hospital in México in 13 years

**DOI:** 10.1093/ofid/ofae631.1607

**Published:** 2025-01-29

**Authors:** Lindsay A Concha-Mora, Aracely Guzman-Guajardo, Pablo D Treviño-Valdez, Jose Eduardo Mares-Gil, Oscar Tamez-Rivera

**Affiliations:** Pediatric Residency Program, Programa Multicéntrico de Especialidades Médicas ITESM- SSNL, Tecnológico de Monterrey. Escuela de Medicina y Ciencias de la Salud. Monterrey, México, Monterrey, Nuevo Leon, Mexico; Tecnologico de Monterrey, Monterrey, Nuevo Leon, Mexico; Tecnologico de Monterrey, Escuela de Medicina y Ciencias de la Salud, Monterrey, Nuevo Leon, Mexico; Pediatric Residency Program, Programa Multicéntrico de Especialidades Médicas ITESM- SSNL, Tecnológico de Monterrey. Escuela de Medicina y Ciencias de la Salud. Monterrey, México, Monterrey, Nuevo Leon, Mexico; Tecnologico de Monterrey, Escuela de Medicina y Ciencias de la Salud, Monterrey, Nuevo Leon, Mexico

## Abstract

**Background:**

The 2022 "TB Global Report" documents a rise of Tuberculosis (TB) cases worldwide and emphasizes the importance of surveillance strategies. Geographic Information Systems (GIS) characterize geo-temporal trends of communicable diseases and assist in decision-making, including resource allocation. The use of GIS in pediatric TB is limited in Mexico, where the incidence of TB among children is high. Mexico’s public health system lacks outpatient clinics (OC) for diagnosis, treatment and prevention of TB. Identifying strategic areas to implement these facilities may reduce the burden of TB.

**Figure:**
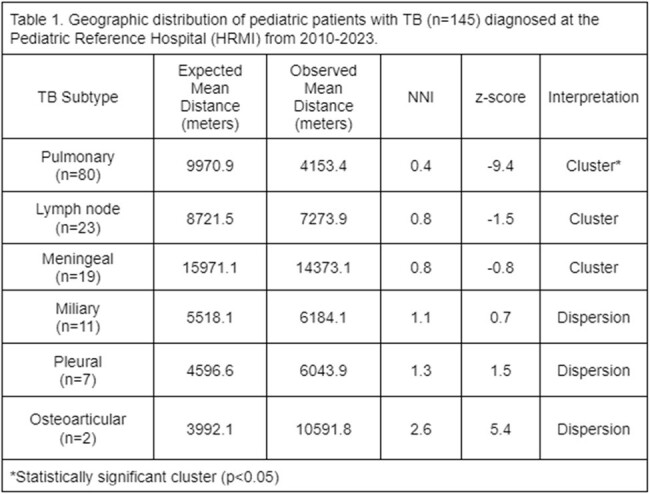

Geographic distribution of pediatric patients with TB (n=145) diagnosed at the Pediatric Reference Hospital (HRMI) from 2010-2023.

Interpretation of NNI and z-score revealed clusttering pattern for pulmonary, lymph node ad meningeal pedaitric TB

**Methods:**

Subjects < 18 years with TB diagnosed at the Pediatric reference hospital (HRMI) in NL Mexico from 2010-2023 were included. Regional marginalization indices were obtained from official local authorities. Distance analysis techniques, nearest neighbor index (NNI) and hierarchical clustering were applied using QGis^®^. Kernel density estimation was calculated, and results were visualized with heat maps.

Map 1. Distribution and Heat Map pf Pediatric TB Cases Diagnosed at the Pediatric Reference Hospital (HRMI) of NL, Mex.

**Figure:**
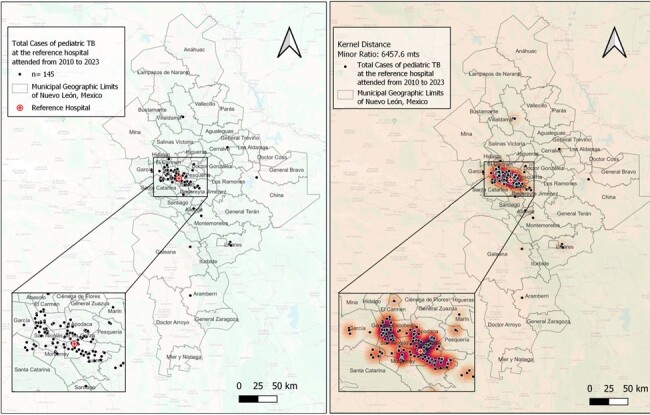

Heat Map was projected by Kernel Density Estimation of 31736.8 meters (SD ± 25279.2 meters), showing hot spots at the Metropolitan Area of Nuevo Leon.

**Results:**

Clinical and geographic data of 145 subjects were included. Three areas showed the highest case concentration. NNI from the total sample showed a dispersion pattern; however, a clustering pattern was observed for pulmonary (p< 0.05), meningeal and lymph node TB (Table 1). Before diagnosis, most patients had at least one visit to a public OC where TB was not suspected. Average distance to the nearest public OC was 0.7 km and to HRMI 21.3 km. Case-to-hospital distance analysis showed that 65% of cases lived >10 km from HRMI. The majority of cases occurred in areas with high (23.5%) and medium (44%) marginalization indices.

Map 2. Heat Maps of Pedaitric TB Subtypes with Clustter Distribution by Kernel Density Estimation (KDE)

**Figure:**
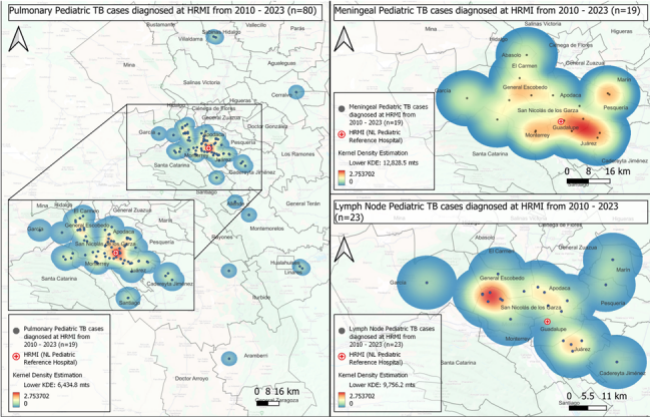

A. Pulmonary Pediatric TB: KDE 6434,8 mts

B. Meningeal Pediatric TB: KDE 12,828 mts

C. Lymph Node Pediatric TB: KDE 9,756 mts

**Conclusion:**

We identify pediatric TB hotspots in NL, Mexico. The clustering patterns identified for pulmonary, lymph node, and meningeal TB indicate a non-random distribution, possibly influenced by marginalization, population density, and healthcare access. Long case-to-hospital distances and failure of general OCs to suspect pediatric TB highlight the importance of implementing specialized TB OCs in strategic high burden areas. Increasing the visibility of pediatric TB and implementing public health strategies according to community needs is crucial. Our full report will be shared with local health authorities in order to propose the creation of TB OCs.

**Figure:**
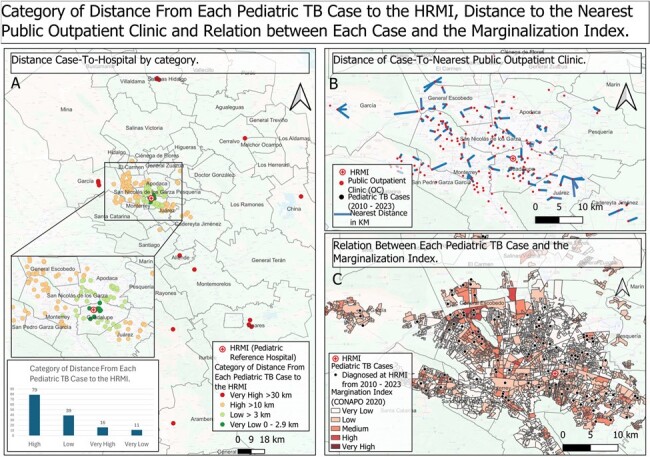

A. Map of distance Case-To-Hospital, category was determine by 4 gropus: Very Low (0 - 2.9 km), Low (3 - 9.9 km), High ( 10 - 29.9 km) and Very High ( > 30 km)

B. Map of distance Case-To-Nearest OC, it demostrates that every case, was less tan 3km from an OC

C. Map of relation of each case location and the Marginalization Index obtained by national authorities, showing vast majority in medium and high index areas

**Disclosures:**

**All Authors**: No reported disclosures

